# Beyond Ebola: surveillance for all hemorrhagic fever in West Africa should be enhanced

**DOI:** 10.11694/pamj.supp.2015.22.1.5837

**Published:** 2015-10-10

**Authors:** Marie Munoz, Valéry Ridde, Seydou Yaro, Carol Bottger

**Affiliations:** 1 School of Public Health (ESPUM), University of Montreal, Montreal, Canada; 2 University of Montreal Public Health Institute (IRSPUM); 3 Ebola Task Force Team / Hemorrhagic Fever Laboratory, Centre Muraz, Bobo-Dioulasso, Burkina Faso

**Keywords:** Ebola, West Africa, diseases surveillance system, Burkina Faso

## Commentary

The Ebola crisis in West Africa has highlighted the vulnerability of individuals and health systems to infectious diseases. For now, the situation appears to be confined to a few countries mainly Guinea, Liberia, Sierra Leone but the entire region is at risk of outbreaks, namely because of cross-border movement. To our knowledge, Ministries of Health of each country have prepared plans for response and communicated their needs to the technical and financial partners. Burkina Faso, for example, prepared in May 2014 a US$ 26 million plan for a period of 12 months which was updated at the end of the year. It is a multi-sectoral plan that aims to 1) strengthen the skills of health care workers (HCW) and other actors, 2) enhance information and awareness of stakeholders, and 3) improve the technical diagnosis capacity of the structures by acquiring a mobile laboratory (type PIII), consumables and reagents. Identification of sites to isolate and treat Ebola cases as well as the creation of specialized teams for secure management of corpses are also planned [1].

Despite all of the preparedness efforts, certain characteristics of the countries epidemiological profile, the local health care and surveillance systems, and also the specific nature of Ebola can hinder efforts to identify cases and respond in a timely fashion. To name a few issues, one needs only to think about: i) the epidemiological pattern of the region that has always been dominated by febrile illnesses; ii) the existence of other hemorrhagic fevers whose specific diagnoses are often difficult to disentangle clinically iii) the unspecific initial symptoms of Ebola which are clinically superimposable on that of several other febrile illnesses [2]. In this context, how will HCWs be able to identify the eventual Ebola case who presents at a front line health facility?

### Febrile illnesses: is it Malaria, Dengue or Ebola? The example of Burkina Faso

As the Ebola epidemic raged in West Africa, our team initiated a research program in Burkina Faso, a country that has not been directly affected so far. Our studies aim to understand different aspects of febrile illnesses such as the professional practices of primary health care workers regarding non-malaria febrile illnesses and the burden of disease caused by dengue in the capital, Ouagadougou. Members of our team have been involved in Ebola response while others have had the opportunity to visit different types of health care facilities and to exchange with several stakeholders from peripheral and central levels such as front line health workers, directors of medical services, as well as different government representatives for neglected tropical diseases, surveillance, malaria programs and World Health Organization. This process has allowed us to gain considerable insight into the challenges that Ebola poses in this country.

Burkina Faso is one of the countries in West Africa where febrile diseases, often caused or attributed to Malaria, account for the majority of health care consultations. In recent years, the contribution of non-malaria febrile illnesses has become increasingly obvious. In 2006, in the western region of the country (Banfora and Bobo), 78% of cases of fever had a negative malaria Rapid Diagnostic Test (RDT) during the dry season and 46% during the rainy season [3]. Since the gradual integration of RDTs to diagnose malaria in 2009, non-malaria fevers have posed a significant clinical challenge for health workers, especially in peripheral health facilities that do not have laboratory facilities.

In 2013, presumed cases of malaria that did not behave in the usual manner drew the attention of the local media and health authorities. Dengue, a disease that was virtually unknown by the public and most HCWs made its appearance on the health scene. The investigation of the outbreak by the Ministry of Health found that 29.7% of suspected cases according to the definition of the National Integrated Surveillance and Response Guide [4] had a positive RDT for dengue [5]. In 2013, our research team reported dengue rates of 8.7% in febrile adults with a negative RDT for malaria in the capital Ouagadougou and 2.7-10% in febrile children in a population-based study in semi-urban areas [6, 7]. There are indications that dengue is endemoepidemic and that the 4 serotypes are in circulation but the situation is too poorly understood for the moment to further describe its epidemiological characteristics such as the incidence, prevalence, seasonality and epidemic peaks [6, 7]. The Department of Health of Burkina Faso has made dengue a priority for 2014 by undertaking a series of actions such as writing a surveillance plan for dengue and preparing training modules for HCWs which should have been ready by the end of 2014. However, these actions have faced challenges such as the lack of funding for diagnostic tests, research and validation meetings. Also since the beginning of the epidemic, attention and efforts have been displaced to provide a greater focus on the Ebola response.

### Surveillance of dengue and hemorrhagic fevers in Burkina Faso

In 2000 Burkina Faso was the first country in West Africa to adopt the integrated surveillance approach recommended by the WHO. An elaborate program targets 45 diseases that are important from a public health perspective [4] with a clearly established pathway that allows sanitary information flow to reach decision making instances at the Ministry of Health from health care facilities effectively. This system has demonstrated its usefulness in the fight against several infectious diseases such as meningitis, dracunculiasis and poliomyelitis.

However, despite the undeniable strengths of the national system, dengue and other hemorrhagic fevers surveillance encounter an array of obstacles. First, HCWs at all levels of training significantly lack knowledge about dengue so it is rarely evoked as a diagnosis. Indeed, preliminary results from a study conducted by our team to evaluate professional practices of HCWs regarding non-malaria febrile illnesses indicate that few of them had received training on dengue or non-malaria febrile illnesses. Furthermore, when presented with clinical vignettes illustrating fever cases with negative malaria RDTs most HCWs chose to treat the patients with antibiotics and few could recall danger symptoms or mentioned dengue.

Frontline health care workers also lack diagnostic means to tell apart the undifferentiated fevers that they encounter. In the past years, dengue RDTs have become available but mainly in the private health sector. They remain financially and geographically inaccessible for a large part of the population. There is only one reference laboratory for hemorrhagic fevers in the country. It has irregular and scarce access to reactives other than for yellow fever. At the present moment it is not qualified to test samples for Ebola. Samples are sent to Dakar or to France to test for a panel of hemorrhagic fever with the delays and costs that this conveys.

Finally, in past years dengue was hardly, if ever, notified. Nevertheless, with the growing attention given to this disease, notification practices in health care settings have evolved rapidly. The center region (which includes the capital) did not report any cases of dengue in 2013 while 479 cases had been reported by October in 2014. Close examination of these figures show that they come from a few clustered health care centers mostly private. Indeed, unequal participation in the reporting at different levels of the health system in particular private facilities, NGOs and hospitals, where it is more likely to find diagnostic means and severe cases, also compounds the problem of patchy surveillance. In addition, hemorrhagic fevers are not reportable as a syndrome. Even if the suspect case definition for Ebola disease is consistent with the description of an hemorrhagic fever and should be reported immediately, there have been none notified in 2014 despite the fact that HCWs have consistently reported cases of fatal hemorrhagic fever caused by dengue.

### Support for Ebola is also empowering countries to distinguish between different febrile syndromes

The main actors on the ground such as MSF and the WHO have made public a series of recommendations to limit the extent of the epidemic. Among other actions, raising awareness about all hemorrhagic fevers, strengthening health systems, especially surveillance, and increasing universal precautions at all levels have been advocated as important actions [8].

The introduction of Ebola in any neighbouring country of those currently affected is sadly a risk that all West African countries must face as happened in Mali and Nigeria. Recognition of the disease based on contact with infected cases could well be ineffective given high mobility in the region. Furthermore, reports of other hemorrhagic fevers in the region for instance the outbreak of Lassa fever in Benin in November 2014 [9] could further complicate the correct identification and management of potential Ebola cases. This has led us to believe that Ebola and other hemorrhagic fevers cannot be considered separately as a vertical problem but need to be taken as a syndromic approach as far as initial management, diagnosis and surveillance are concerned. We are joining our voices with those who advocate for strengthening health systems as a whole, and not only services targeting Ebola specifically. Supporting the efforts of the Ministries of Health in the region to increase the ability to diagnose and manage hemorrhagic fever is thus paramount. Health facilities at all levels need to be provided with individual and collective means of protection that can be used from the first contact with the patient onwards as pointed out by the WHO in directives for malaria management [10]. Front line HCWs must be trained to diagnose and manage the different febrile illnesses in particular hemorrhagic fevers and this cannot be achieved without making available appropriate diagnostics means. Research for RDTs for Ebola, as pointed out by the WHO, is of course essential [11], as is the availability of dengue RDTs and reactives to test a panel of hemorrhagic fevers in national decentralized laboratories. Finally, the monitoring system of all hemorrhagic fevers, not only Ebola, needs to be supported. It is also time to think about innovative solutions that integrate current and new information technologies to enable existing systems to improve the detection of infectious disease and enable timely response (Geographical Information systems (GIS), mobile phones, open access softwares, new collaborations between academic sectors and governments).

Burkina Faso and other countries in the West African region are preparing their response for a possible introduction of Ebola. In a context where febrile illnesses are already the main reason for consultation, creating systems that have the ability to distinguish between the different patterns of fevers in a timely fashion, especially hemorrhagic fever, appears to be vital to protect healthcare workers and to contain new outbreaks in yet unaffected countries.
